# Functional Diversification of the Dihydroflavonol 4-Reductase from *Camellia nitidissima* Chi. in the Control of Polyphenol Biosynthesis

**DOI:** 10.3390/genes11111341

**Published:** 2020-11-12

**Authors:** Lina Jiang, Zhengqi Fan, Ran Tong, Xingwen Zhou, Jiyuan Li, Hengfu Yin

**Affiliations:** 1 State Key Laboratory of Tree Genetics and Breeding, Research Institute of Subtropical Forestry, Chinese Academy of Forestry, Hangzhou 311400, China; jianglinayls@163.com (L.J.); fzq_76@126.com (Z.F.); tongranyls@126.com (R.T.); 2 Key Laboratory of Forest Genetics and Breeding, Research Institute of Subtropical Forestry, Chinese Academy of Forestry, Hangzhou 311400, China; 3 College of Biology and Pharmacy, Yulin Normal University, Yulin 537000, China; xingwenzhou2003@163.com

**Keywords:** floral pigmentation, dihydroflavonol 4-reduetase, *Camellia nitidissima* Chi., polyphenols, anthocyanins

## Abstract

Plant secondary metabolism is complex in its diverse chemical composition and dynamic regulation of biosynthesis. How the functional diversification of enzymes contributes to the diversity is largely unknown. In the flavonoids pathway, dihydroflavonol 4-reductase (DFR) is a key enzyme mediating dihydroflavanol into anthocyanins biosynthesis. Here, the *DFR* homolog was identified from *Camellia nitidissima* Chi. (*CnDFR*) which is a unique species of the genus *Camellia* with golden yellow petals. Sequence analysis showed that CnDFR possessed not only conserved catalytic domains, but also some amino acids peculiar to *Camellia* species. Gene expression analysis revealed that *CnDFR* was expressed in all tissues and the expression of *CnDFR* was positively correlated with polyphenols but negatively with yellow coloration. The subcellular localization of CnDFR by the tobacco infiltration assay showed a likely dual localization in the nucleus and cell membrane. Furthermore, overexpression transgenic lines were generated in tobacco to understand the molecular function of *CnDFR*. The analyses of metabolites suggested that ectopic expression of *CnDFR* enhanced the biosynthesis of polyphenols, while no accumulation of anthocyanins was detected. These results indicate a functional diversification of the reductase activities in *Camellia* plants and provide molecular insights into the regulation of floral color.

## 1. Introduction

Plants produce multifarious secondary metabolites that play pivotal roles in various aspects related to development, growth and survival. Flavonoids (including polyphenolic compounds) are a major class of secondary metabolites that are produced in diverse plant lineages; they play an important role in the coloration and resistance of plants [1,2,3]. Many specific chemicals of flavonoids from plants (e.g., tea, grape and cacao) are found to be beneficial to human health [4,5,6]. Currently, the biosynthesis pathway of flavonoids is extensively characterized; at the initial step, phenylalanine is catalyzed to 4-coumaryol-CoA to generate chalcones which are backbones of flavonoids [7,8]. After multiple steps of enzymatic reaction, flavones, flavonols, polyphenols and anthocyanins are finally synthesized [9,10,11].

Dihydroflavonol 4-reductases (DFRs) are key enzymes involved in the production of anthocyanins from dihydroflavanol [12,13,14]. DFRs use NADPH as a cofactor to catalyze the dihydroflavanols (including dihydrokaempferol DHK, dihydroquercetin DKQ, dihydromyricetin DHM) to generate colorless leucoanthocyanidins (leucopelargonidin, leucocyanidin and leucodelphinidin), which is a critical step of floral pigments biosynthesis [15,16]. The leucoanthocyanidins can be further converted to anthocyanin by anthocyanin synthase (ANS) and UDP-flavonoid glucosyltransferase (UFGT) that are major floral pigments [17,18,19]. Meanwhile, leucoanthocyanidins can also be directed to the polyphenol biosynthesis (catechin and epcatechin) by leucocyanidins reductase (LAR) and anthocyanin reductase (ANR) [20,21,22].

DFR of different species has specific selectivity for three dihydroflavanols (DHK, DHQ and DHM), so that the metabolic pathway of anthocyanin is carried out in different directions, leading to different flower colors in plants [23,24]. The catalytic property of DFRs has been found to be a limiting factor of floral color variation [25,26,27]. For instance, Johnson and coauthors have shown that minor changes of DFR sequences can result in substrate specificity and the pelargonidin-type anthocyanins lacking consequently [28]. Several *DFR* genes have been isolated and identified, and their functions have also been studied, including *Camellia sinensis* L. [29], *Dendrobium* [30], *Saussurea medusa* Maxim. [31], *Agapanthus praecox* [32], *Maluscrabapples* [14] and *Vitisbellula* [33]. The functions of *DFR* genes in these plants have been shown to be related to anthocyanin synthesis. The mutants of *DFR* in *Ipomoea nil*, generated by the CRISPR/Cas9 technology, display white flowers with a great loss of floral anthocyanins [34].

*DFR* of *Camellia sinensis* L. has different functions to other plants. Overexpression of *CsDFR* in tobacco elevates levels of polyphenols and enhances stress resistance, for example, the free radical scavenging activity was improved in transgenic tobacco and transgenic lines showed resistance against drought stress, oxidative stress, abscisic acid and infestation by a tobacco leaf cutworm *Spodoptera litura* [29,35]. However, the roles of *DFRs* of *Camellia* species in the regulation of floral pigmentation are still unknown. *Camellia nitidissima* Chi. is known as the “Queen of the tea tribe” due to the golden-color flowers. Studies reveal that the yellow coloration of *C. nitidissima* Chi. petals is mainly from the quercetin derivatives [36,37,38], and accumulation of aluminum ions and pH are found to be key factors to induce the yellow coloration [39,40]. We hypothesize that the functional diversification of the DFR homolog from *C. nitidissima* Chi. is involved in the regulation of flavonoid biosynthesis that contributes to petal pigmentation. Recently, transcriptomes analyses were performed in *C. nitidissima* Chi. and related yellow *Camellia* species; genes involved in the biosynthesis pathway of secondary metabolism were uncovered [41,42]. Functional analysis of flavonol synthase in *C. nitidissima* Chi. (*CnFLS*) has indicated that the regulation of the bifurcation of secondary metabolism plays a key role in the floral pigmentation [41,42]. Previous studies in other plants have also found that the competition between *FLS* and *DFR* can cause the change in floral color [16,43,44]. Here, the *DFR* homolog from *C. nitidissima* Chi. (*CnDFR*) is identified and characterized, and its roles in the regulation of the secondary metabolism pathway are revealed. This work provides a theoretical basis for the application of *DFR* in flower color cultivation in ornamental Camellias.

## 2. Materials and Methods

### 2.1. Plant Materials and Growth Conditions

*Camellia nitidissima* Chi. tissues were collected from the National Camellia Germplasm Resource Bank (Guangxi, China, E 108°20′53′′ N 22°49′11′′, 75 m above sea level) in Nanning, Guangxi province, China. The materials were frozen with liquid nitrogen and stored at −80 °C for later use.

*Nicotiana benthamiana* Domin was used for the transient transformation and stable transformation experiments. The seedlings were grown in a growth chamber (RDN-1000 E, Ningbo Yang hui Instrument Co. Ltd., Ningbo, China; temperature: 25 °C, humidity: 76%, illumination: 6000 Lx, light cycle: 16/8 h).

### 2.2. Cloning of CnDFR

Total RNAs were isolated using RNAprep Pure Extraction Kit (DP441, TIANGEN biochemical Technology, Beijing, China) and we determined the integrity of RNA based on the results of the 1.5% agarose gel electrophoresis analysis. According to the instructions of PrimeScript II 1st Strand cDNA Synthesis Kit (6210, TaKaRa, Tokyo, Japan), we synthesized the cDNA for gene cloning experiments. We designed a pair of specific primers (Table S1. 1) by Primer 3 (http://www.primer3plus.com/cgi-bin/dev/primer3plus.cgi) according to the transcriptome data. PCR products were obtained, and we cloned them into a T-vector (CT501, TransGen Biotech Co., Ltd., Beijing, China) for sequencing. The full length of *DFR* was assembled and verified based on sequence analysis.

### 2.3. Sequence Alignment and Phylogenetic Analysis

The BioEdit software and NCBI Blast online (https://blast.ncbi.nlm.nih.gov/Blast.cgi) were used to align the sequences [45]. We used NCBI ORFfinder (http://www.ncbi.nlm.nih.gov/projects/gorf/) to find reading frames [46]. Protparam online (https://web.expasy.org/protparam/) was used to analyze protein molecular weight and isoelectric point and so on [47]. Meanwhile, amino acid sequence alignment was performed by DNAMAN and a phylogenetic tree was constructed with MEGA 5.0 software, using the neighbor-joining (NJ) method and 1000 bootstrap replicates [48].

### 2.4. Quantitative PCR Analysis of CnDFR

*GAPDH* as the reference gene (Table S1. 2) was used for quantitative PCR analysis (Table S1. 3). Using PrimeScript RT reagent Kit with gDNA Eraser (RR047, TaKaRa, Japan), the cDNA first strand was synthesized. Using SYBR Prime Ex Tap II (Tli RNaseH Plus) (RR420, TaKaRa, Japan), the quantitative PCR analysis was performed according to the user’s manual. The reaction was performed on QuantStudio^®^ 7 Flex (Applied Biosystem, Foster City, CA, USA) and the reaction procedure was as follows: pre-denaturation at 95 °C for 30 s; 98 °C 5 s, 60 °C 34 s, 40 cycles; 95 °C 15 s, 60 °C 1 min, 95 °C 15 s. The relative expression level of *CnDFR* in different organs and different development periods was measured with *GAPDH* as the housekeeping gene by the 2 ^(−ΔΔCT)^ method [49].

### 2.5. High-Performance Liquid Chromatography Analysis

The NF555 colorimeter (Nippon Denshoku Industries Co., Ltd., Tokyo, Japan) was used to detect the color indicator of petals. HPLC analysis was performed to measure the flavonoids, polyphenols and anthocyanins constituents. We grinded a fresh sample of 0.6 g weight in liquid nitrogen, supplied with 5 mL extraction solution (methanol/water/formic acid/trifluoroacetic acid = 70:27:2:1). Then, we extracted in the dark for 24 h, shaking in the middle a few times. After extraction, we filtered the sample with absorbent cotton to remove residue and pass it through the organic microporous filter membrane (0.22 cm) (ANPEL Laboratory Technologies (Shanghai) Inc., Shanghai, China). The filtrate was used in the machine analysis.

We used Agilent Technologies 1260 Infinity (Agilent Technologies, Inc., Waldbronn, Germany) and Waters SunFire C18 column (4.6 × 250 mm, 5 μm) (Waters Co., Belleville, IL, USA) for the HPLC analysis. The column temperature was 30 °C. The flow rate was 1.0 mL/min, and the injection volume was 10 μL. The elution mobile phases were (A): 2% formic acid solution, and (B): pure acetonitrile. The elution procedure for flavonoids: 0–5 min, 20% B; 5–15 min, 20% up to 40% B; 15–20 min, 40% up to 60% B; 20–20.2 min, 60% down to 20% B; 20.2–24 min, 20% B. The detection wavelength of flavonoids was 350 nm. The elution procedure for polyphenols: 0–9 min, 98% down to 90.7% B; 9–15 min, 90.7% B; 15–20.5 min, 90.7% down to 85% B; 20.5–29.5 min, 85% down to 75% B; 29.5–30 min, 75% up to 98% B; 30–34 min, 98% B. The detection wavelength of polyphenols was 278 nm.

### 2.6. Agroinfiltration-Based Transient CnDFR-EGFP Gene Expression in Nicotiana Benthamiana Domin for Subcellular Localization of CnDFR

We designed a pair of primers (Table S1. 4) according to the EXclone Kit instructions (exv09, Hangzhou Biogle Co. Ltd., Hangzhou, China) for the vector construction. The overexpression vector was transformed to the *Agrobacterium GV3101* strain by the thermal shock method [50]. To perform tobacco infiltration analysis, the transformed agrobacterium was suspended using induction medium (10 mM/L MES + 10 mM/L MgCl_2_ + 100 uM/L AS) and injected into the *Nicotiana benthamiana* Domin leaf [51]. Then, the GFP signals were detected 2~5 days after injection by a LSM510 Meta device (Zeiss, Jena, Germany) [52].

### 2.7. Tobacco Transformation Analysis of CnDFR

To verify the functionality of *CnDFR*, we performed transformation of *Nicotiana benthamiana* Domin using the leaf plate method [53]. We used T5 Direct PCR Kit (Plant) (TSE011, TSING KE Biological Technology, Tianjin, China) for positive identification of rooting plants with PCR primers (Table S1. 5). PCR procedure was as follows: pre-denaturation at 98 °C for 3 min, denaturation at 98 °C for 10 s, annealing at 65 °C for 10 s, extension at 72 °C for 1 min and 30 s, 30 cycles, extended at 72 °C for 5 min and detected by 1% agarose gel electrophoresis. After the positive plants flowering, we collected the flowers, froze them with liquid nitrogen and stored them at −80 °C. The quantitative PCR was performed to measure the relative expression of *CnDFR* and determine total flavonoids, total polyphenols and total anthocyanins in flowers by a spectrophotometer [39]. Constituents of flavonoids, anthocyanin and polyphenols in flowers were also determined by HPLC and compared with the control group.

## 3. Results

### 3.1. Molecular Characterization of CnDFR Reveals Lineage-Specific Amino Acid Sites

**Figure 1:**
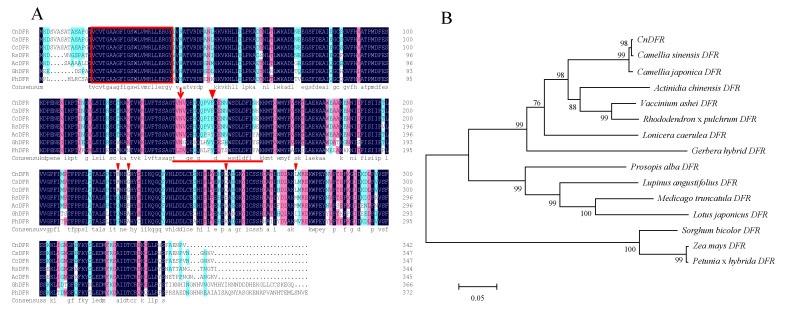
Amino acid alignment and phylogenetic analysis of the homolog plant DFRs of *Camellia nitidissima* Chi. (CnDFR). (**A**) Alignment of DFR-like protein sequences. CnDFR, *Camellia nitidissima* DFR; CsDFR, *Camellia sinensis* DFR; CcDFR, *Camellia chekiangoleosa* DFR; RsDFR, *Rhododendron simsii* DFR; AcDFR, *Actinidia chinensis* DFR; GhDFR, *Gerbera hybrida* DFR; PhDFR, *Petunia hybrida* DFR. The red boxed region is a putative NADPH-binding region. The region underlined is predicted to be the substrate specificity-determining region. At the 134 th amino acid, there is an asparagine (N) different from *Petunia* but identical to *Gerbera* and so on. The red triangle is the different amino acids of *Camellia* DFRs from other plants. (**B**) Phylogenetic tree of *CnDFR* constructed with MEGA 5.0, using the neighbor-joining (NJ) method and 1000 bootstrap replicates. *CnDFR* sequence was found to be 75% to 99% similar to homological *DFR* genes.

Based on transcriptome sequences of *C. nitidissima* Chi. [41], the full-length CDSsequence of *CnDFR* was obtained through gene-specific amplification (GenBank accession number MN276188). The *CnDFR* transcript encoded a protein of 342 amino acids, with a NADPH binding site of 21 amino acids (VTGAAGFIGSWLVMRLLERGY; Figure 1A). We performed sequencing alignment analysis of CnDFR with other homologs from various plant species. Then, it was found that all sequences included the conserved NADPH binding motif at the N terminal, and the substrate specificity-determining amino acid was identical in all other species but different from *c hybrida* L. (Figure 1A). In the substrate specificity-determining region, there was a phenylalanine (F) in all DFRs of *Camellia* species, different from other plants (Figure 1A); there were an additional four amino acids that were unique to *Camellia* species (Figure 1A), which may result in different functions of *Camellia* DFRs.

To evaluate the phylogenetic relationships of *DFR*s, we aligned 14 *DFR* sequences from various plant species to construct a phylogenetic tree (Figure 1B). The results showed that the three *Camellia DFRs* formed a clade together that was close to *Actinidia chinensis* (Figure 1B). These results suggested that *CnDFR* was potentially functionally conserved in the secondary metabolism pathway, and *DFRs* from *Camellia* species might have unique functions compared to other plants.

### 3.2. Expression of CnDFR in C. nitidissima Chi. Is Positively Correlated with Polyphenols Contents

**Figure 2:**
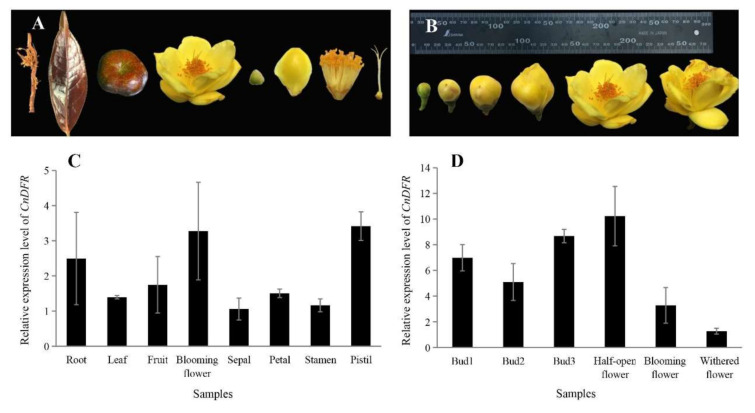
Relative expression level of *CnDFR* in *C.nitidissima* Chi. (**A**) Tissues of *C.nitidissima* Chi.: root, leaf, fruit, flower, speal, petal, stamen, pistil. (**B**) Flowers at different developmental stages of *C.nitidissima* Chi.: bud in 10 mm, bud in 20 mm, bud in 30 mm, half-open flower, blooming flower, withered flower. (**C**) Relative expression level of *CnDFR* in different tissues of *C.nitidissima* Chi. *CnDFR* expressed in all of the tissues, while the expression was the highest in the flower and the lowest in the sepal. (**D**) Relative expression level of *CnDFR* in flowers at different developmental stages. The expression of *CnDFR* showed a trend of first decreasing then increasing and then decreasing, and the expression was the highest in the half-open flower.

To study the expression profiles, the quantitative PCR (qPCR) analysis of *CnDFR* in different tissues of *C. nitidissima* Chi. was performed with *GAPDH* as the housekeeping gene (Figure 2A,B). It is found that *CnDFR* expressed in all tissues including the root, leaf, fruit, flower, sepal, petal, stamen and pistil (Figure 2C). During flowering, the expression of *CnDFR* is maintained at a high level in floral bud differentiation stages (Figure 2D) and reaches the highest expression when the flowers are half open, and then decreases rapidly after the blooming stage (Figure 2D).

**Figure 3:**
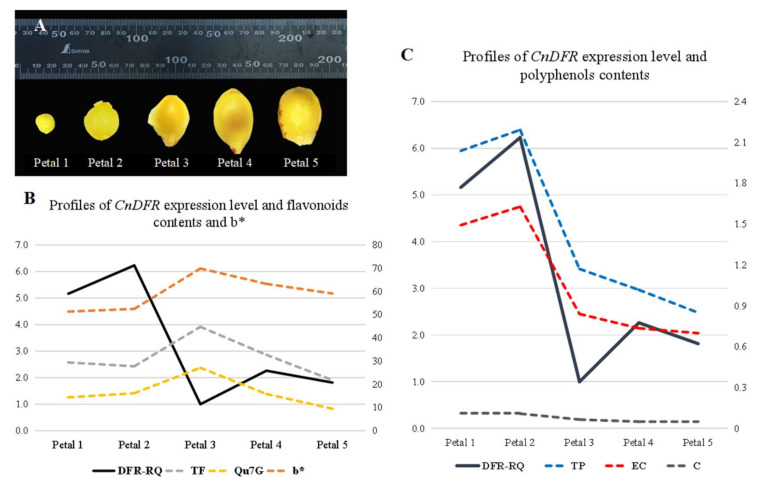
The relationship between *DFR* relative expression level and the chemical components content in petals of *C. nitidissima* Chi. (**A**) Petals in 5 stages of *C. nitidissima* Chi.: petals of young bud, petals of big bud, petals of half-open flower, petals of blooming flower, petals of withered flower. (**B**) *CnDFR* relative expression level and flavonoids contents and b* in petals of *C. nitidissima* Chi. DFR-RQ (the relative expression level of *DFR*) in 5 stages had an M-shaped trend. TF (the content of total flavonoids), Qu7 G (quercetin-7-O-β-D- glucopyranoside) and b* (yellow color index of petals) were negatively correlated with the expression level of *CnDFR*. (**C**) *CnDFR* expression level and polyphenols components. TP (the content of total polyphenols), EC (the content of epicatechin) and C (the content of catechin) were positively correlated with the expression level of *CnDFR*.

*DFR* genes play a key role in the biosynthesis of plant secondary metabolites. In order to study the relationship between *CnDFR* and flower color in *C. nitidissima* Chi., the yellow color index b * and content of flavonoids and polyphenols were determined in the petals in five stages of *C. nitidissima* Chi., and we analyzed their relationships to the expression of *CnDFR* (Figure 3). By correlation analysis, it is found that the relative expression of *CnDFR* (DFR-RQ) is negatively correlated with TF (the content of total flavonoids), Qu7 G (quercetin-7-O-β-D- glucopyranoside) and b* (yellow color index of petals). However, it is positively correlated with the contents of total polyphenols and some components of polyphenols (Figure 3B). The results indicate that a high level of *CnDFR* expression is correlated with enhanced polyphenols biosynthesis and potentially causes the yellow color to become lighter in *C. nitidissima* Chi. (Figure 3C).

### 3.3. Subcellular Localization of CnDFR Was in the Nucleus and Cell Membrane

**Figure 4:**
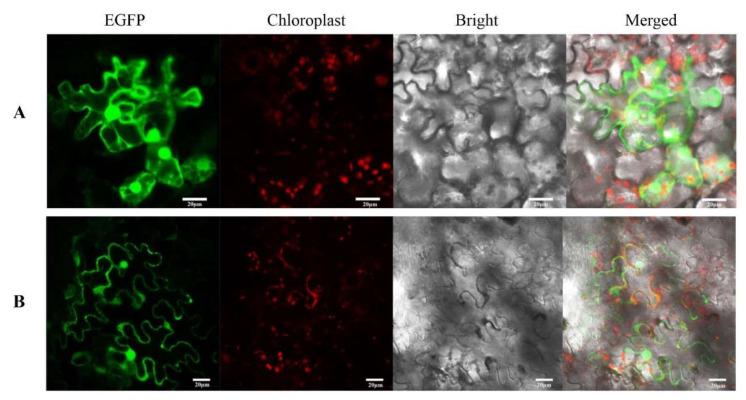
The subcellular localization of CnDFR. (**A**) Observation by LSM510 Meta of the EGFP empty vector. White scale: 20 µm. The green fluorescence signals appeared in the nucleus, cell membrane and cytoplasm under the excitation of the wavelength of 488 nm. (**B**) Observation of the lower epidermal cells of *Nicotiana benthamiana* leaves with the CnDFR-EGFP vector. White scale: 20 µm. The nucleus and cell membrane expressed a strong green fluorescence signal.

To investigate the subcellular localization of CnDFR, we performed transient expression analysis using tobacco infiltration (Figure 4). A constitute expression construct harboring the fusion protein of CnDFR with green fluorescent protein (EGFP) was introduced into tobacco leaf. We found that free EGFP signals appeared in the nucleus, cell membrane and cytoplasm, and the signals were scattered throughout the whole cell (Figure 4A). Meanwhile, the signals of CnDFR-EGFP were found to be localized in the cell membrane as well as the nucleus (Figure 4B), suggesting an extremely likely dual subcellular localization of CnDFR.

### 3.4. Overexpression of CnDFR Enhanced the Biosynthesis of Polyphenols But Not Anthocyanins

**Figure 5:**
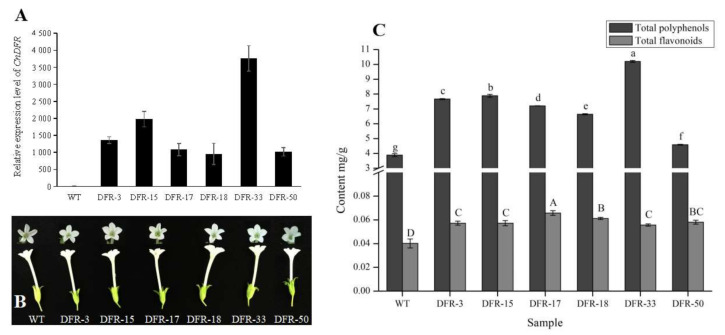
Relative expression of *CnDFR* and contents of total flavonoids, total polyphenols and total anthocyanins in flowers of transgenic tobaccos. (**A**) The expression of *CnDFR* in tobacco flowers. *CnDFR* expression of transgenic lines was significantly higher than the wild type. (**B**) Flowers of wild type and transgenic tobaccos. The flowers of wild type and transgenic strains were white with no significant difference. (**C**) The contents of total polyphenols and total flavonoids. Total polyphenols in most of transgenic strains were about twice the wild type. Total flavonoids were very low overall, and the contents in transgenic strains were 1.5 times the wild type. The letters represent the level of difference.

To study the biochemical functions of *CnDFR*, we generated overexpression lines using transgenic tobacco. Six transgenic lines with positive resistance were validated using construct-specific primers (Table S1, Figure S1). Further, we measured the relative expression levels of *CnDFR* in transgenic tobacco lines; the results showed that all tested lines displayed ectopic expression of *CnDFR* compared to the wild type plant (Figure 5A), while no visible phenotypes were observed in the transgenic lines (Figure 5B).

Meanwhile, the contents of the secondary metabolites in flowers, including flavonoids, polyphenols and anthocyanins, were determined. It is found that there is no total anthocyanins content detected and the total polyphenols are significantly higher in transgenic lines than those in the wild type (Figure 5C). Besides, the contents of flavonoids are increased slightly and the content of total flavonoids is much lower than total polyphenols (Figure 5C).

**Figure 6:**
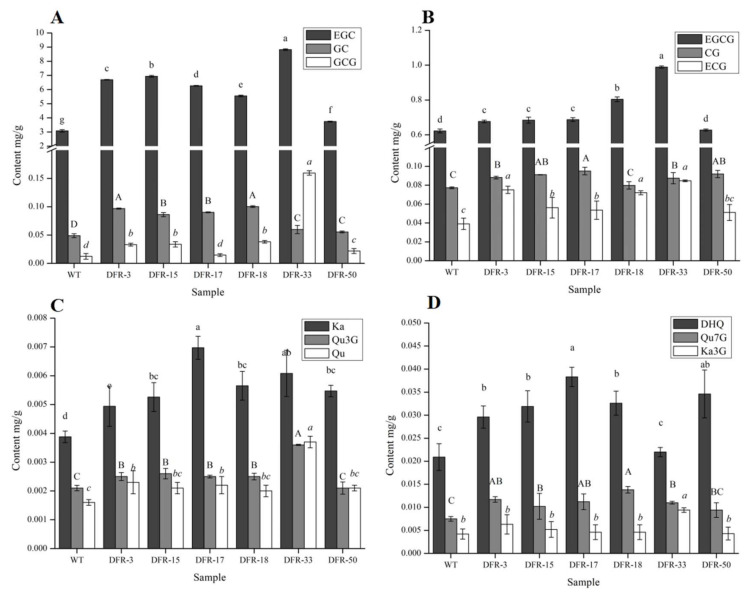
The content of flavonoids and polyphenols in flowers of transgenic tobaccos. (**A**) The content of EGC (epigallocatechin), GC (gallocatechin) and GCG (gallocatechin gallate) in flowers of tobaccos. (**B**) The content of EGCG (epigallocatechin gallate), CG CG (catechin gallate) and ECG (epicatechin gallate) in flowers of tobaccos. The contents of EGC, GC, GCG (except DFR-17), EGCG (except DFR-50), CG (except DFR-18) and ECG (except DFR-50) in the positive lines were significantly higher than those in the wild type tobacco except one line. (**C**) The content of Ka (kaempferol), Qu3 G (quercetin-3-O-glucopyranoside) and Qu(quercetin) in flowers of tobaccos. (**D**) The content of DHQ (dihydroquercetin), Qu7 G (quercetin-7-O-β-D-glucopyranoside) and Ka3 G (kaempferol-3-glucopyranoside) in flowers of tobaccos. DHQ (except DFR-33) and Ka in the positive lines were significantly higher than those in the wild type tobacco. The contents of flavonols (Qu3 G, Qu7 G and Ka3 G) have no significant change between the positive lines and the wild type. The letters represent the level of difference.

In order to understand the compositional changes of secondary metabolites, the contents of different chemicals were measured by HPLC. Six flavonoid and six polyphenol standards were used to reveal the changes in contents in transgenic lines. We detected that the EGC and GC were significantly higher than the wild type in all six transgenic lines (Figure 6A), while GCG, EGCG, CG and ECG were significantly increased in five transgenic lines (Figure 6B). In most of the transgenic plants, the flavonoids contents (including Qu7 G, Qu3 G, Ka3 G, DHQ and Qu) were not significantly different from the wild type (Figure 6C,D), and only the contents of Ka were significantly higher in all transgenic lines (Figure 6C). This indicates that enhanced expression of *CnDFR* promotes the biosynthesis of polyphenols extraordinarily.

## 4. Discussion

The DFR enzymes are key players in the formation of plant pigments and antioxidative flavonoids [23]. The functions of DFRs in different plant lineages have been found to be conserved, which catalyze the reduction step of dihydroflavonols (including DHK, DKQ and DHM) [14,16]. In plants with red anthocyanins, the *DFR* gene is recognized as the first enzyme committed to anthocyanin biosynthesis, after the common phenylpropanoid pathway [28,54]. However, the diversity of the substrate specificity of DFRs is also revealed in some plant lineages [15,27,28]. In this study, we showed that CnDFR possessed conserved catalytic domains and some amino acids specific within *Camellia* species. The sequences of DFR from the genus *Camellia* were highly similar; the phylogenetic analysis also demonstrated that *CnDFR* was closely related to *Camellia* (Figure 1). Meanwhile, we have discovered that there was a conserved phenylalanine (F) of *Camellia* DFRs that differed from other plants in the substrate specificity-determining region (Figure 1). This indicates that *Camellia* DFRs might have different catalytic functions.

Many studies have shown that the expression patterns of *DFR*s have certain tissue specificity and are related to their functions. For instance, Nakatsuka et al. [26] found the *DFR* gene of the Asiatic hybrid was largely expressed in the colored tepals, anthers, filaments, pistils and red scales, while the expression was not detected in the uncolored tissues of the yellow variety. It is shown that, in *C. nitidissima* Chi., *CnDFR* is broadly expressed in various tissues including roots, leaves, fruits and flowers (Figure 2). Detailed analysis during floral development has revealed that the expression pattern of *CnDFR* is positively correlated with polyphenol accumulation (Figure 3). This result also suggests that *CnDFR* is not a determinant directing the anthocyanin biosynthesis in *C. nitidissima* Chi. The yellow pigments in *C. nitidissima* Chi. have been identified majorly as quercetin derivatives [40] belonging to flavonoids. The expression profile of *CnDFR* during floral petal development was positively correlated with polyphenols but negatively with yellow coloration. Therefore, the roles of *CnDFR* in the regulation of the floral color of *C. nitidissima* Chi. need to be further characterized. Through the subcellular localization analysis, we have shown that CnDFR is likely to localize in the nucleus and cell membrane (Figure 4). This result is different from the analysis of *Vitisbellula*, which found VbDFR was mainly located in the cytosol of onion epidermal cells [33].

In the overexpression analysis, it is found that there are no anthocyanins in the flowers of transgenic tobacco lines (Figure 5). These results are not consistent with analyses from other plants. For example, overexpression of *DFR* of *Agapanthus praecox* into *Petunia hybrida* “W85” resulted in a change in floral color from white to fuchsia [32]. Further, down-regulation of *DFRs* from *Nicotiana tabacum* and *Petunia hybrida* reduced the anthocyanin contents and changed the floral color from pink to light pink and white [25,55]. All these studies indicate that *DFR* plays a key role in promoting the formation of anthocyanin and changing floral color, which is different from *CnDFR*. In overexpression lines of *CnDFR*, no anthocyanins were detected, which was also probably related to the transformation plants (*Nicotiana benthamiana*) with white flowers. The flavonoid pathway in *N. benthamiana* may be interrupted and does not have a complete synthesis pathway, leading to less synthesis of the final colored products and no color rendering, which need to be further researched. However, we found that a lot of polyphenols were accumulated in transgenic positive tobacco lines (Figure 6). This indicates a functional diversification of the molecular function of *CnDFR*.

Studies in *C. sinensis* have shown that overexpression *CsDFR* enhanced the biosynthesis of polyphenols and stress resistance of plants [29,35], which supported a functional conservation in *Camellia* species. However, *C. nitidissima* Chi. is unique in its yellow floral color, and the petals of *C. nitidissim* Chi. accumulated a high level of flavonoids. Since most *C. sinensis* species bear white petals, it is not known if *CnDFR* has specified functions of flavonoid biosynthesis that is related to the yellow pigments. Future studies comparing different DFRs from several *Camellia* species might be required to investigate the molecular functions of DFRs.

## 5. Conclusions

We identified the *DFR* homolog from *C. nitidissima* Chi. (*CnDFR*), and its sequence analysis showed that CnDFR possessed some amino acids peculiar to *Camellia* species, among which a specific phenylalanine (F) was observed in *Camellia* DFRs in the substrate specificity-determining region. Phylogenetic analysis showed that *DFRs* of *Camellia* species formed a clade that was close to *Actinidia chinensis*. Gene expression analysis revealed that the expression of *CnDFR* was positively correlated with polyphenols but negatively with yellow coloration. Subcellular localization of CnDFR showed a likely dual localization in the nucleus and cell membrane. Furthermore, in the transgenic tobaccos, it was found that ectopic expression of *CnDFR* enhanced the biosynthesis of polyphenols, while no accumulation of anthocyanins was detected. These results suggest a functional diversification of DFR activities in *Camellia* plants and provide molecular insights into the regulation of floral color.

## Supplementary Materials

The following are available online at https://www.mdpi.com/2073-4425/11/11/1341/s1, Figure S1: PCR identification for positive tobaccos, Table S1: Primer list and application.
