# Supplementary material for: Functional Diversification of the Dihydroflavonol 4-Reductase from Camellia nitidissima Chi. in the Control of Polyphenol Biosynthesis
Source: Genes (Basel). 2020 Nov 12;11(11):1341. doi: 10.3390/genes11111341 (PMC7696568; doi:10.3390/genes11111341)
Supplement: Supplementary file 1 [file genes-11-01341-s001.zip › Supplementary File/Table A1.docx]

**Table A1. Primer list and application**

| **Number** | **Primer name** | **Sequence** | **Application** |
| --- | --- | --- | --- |
| 1 | pDFR-F | TGGAATGGTGAGTGGAGCAC | CDS amplification |
|  | pDFR-R | CACCACCCCACCATTTTTGG |  |
| 2 | GAPDH-F | GGGAATCCTTGGTTACACTGAG | Reference for quantitive PCR |
|  | GAPDH-R | ACCCCATTCGTTGTCATACC |  |
| 3 | DFR-F | ACTGTGGAAGGCGGATTTGA | Quantitive PCR |
|  | DFR-R | CGTTGATTGTCGGCTTGATTAC |  |
| 4 | EXS1 | TCAGCAGTCGAAGAGCATGACTGATACAGTGCGCCC | Overexpression vector construction |
|  | EXS2 | TTAGCGTGTGAAGAGCTATTTGAACCTTGTTGCCA |  |
| 5 | iDFR-F | ATTTGGAGAGAACACGGGGG | Positive identification |
|  | iDFR-R | TTAGCGTGTGAAGAGCTATTTGAACCTTGTTGCCA |  |
