# Supplementary figures and images for: Functional Diversification of the Dihydroflavonol 4-Reductase from Camellia nitidissima Chi. in the Control of Polyphenol Biosynthesis
Source: Genes (Basel). 2020 Nov 12;11(11):1341. doi: 10.3390/genes11111341 (PMC7696568; doi:10.3390/genes11111341)

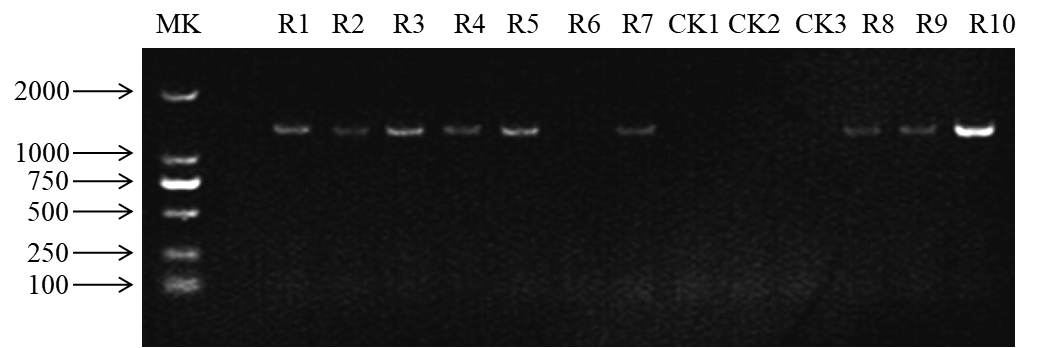

Supplement: Supplementary file 1 [file genes-11-01341-s001.zip › Supplementary File/Figure A1 PCR identification for positive tobaccos.jpg]
